# The Common Haplotype GATGACA in Surfactant-Protein B Gene Is Protective for Respiratory Distress Syndrome in Preterm Neonates

**DOI:** 10.3389/fped.2022.851042

**Published:** 2022-05-25

**Authors:** Silvia Mikolajcikova, Zora Lasabova, Veronika Holubekova, Maria Skerenova, Jana Zibolenova, Katarina Matasova, Mirko Zibolen, Andrea Calkovska

**Affiliations:** ^1^Clinic of Neonatology, Jessenius Faculty of Medicine, Comenius University and University Hospital, Martin, Slovakia; ^2^Department of Molecular Biology and Genomics, Jessenius Faculty of Medicine, Comenius University, Martin, Slovakia; ^3^Biomedical Centre Martin, Jessenius Faculty of Medicine, Comenius University, Martin, Slovakia; ^4^Department of Public Health, Jessenius Faculty of Medicine, Comenius University, Martin, Slovakia; ^5^Department of Physiology, Jessenius Faculty of Medicine, Comenius University, Martin, Slovakia

**Keywords:** haplotype, *SFTPB*, surfactant, respiratory distress, neonate

## Abstract

**Background:**

Respiratory distress syndrome (RDS), a disorder of primary surfactant deficiency resulting in pulmonary insufficiency, remains a significant problem for preterm neonates. Associations between genetic variants of surfactant proteins and RDS have been reported, but haplotypes of the surfactant protein B gene (*SFTPB*) have not been studied. The aim of the study was to prove the hypothesis that certain haplotypes of *SFTPB* may be protective or risk factors for RDS.

**Methods:**

The study was performed with 149 preterm infants, born <34 weeks of gestation, with 86 infants with mild RDS or without RDS (control group) and 63 infants with severe RDS (patient group). RDS was considered severe if multiple doses of exogenous surfactant and/or mechanical ventilation within the first 72 h of life were needed. The venous blood sample was used for the analysis of gene polymorphisms associated with RDS, genotyping, and haplotype estimation. Multivariate logistic regression analysis and the odds ratio were calculated to detect the contribution of the studied variables to the development of RDS.

**Results:**

A new association of the common single nucleotide polymorphism (SNP) rs2304566 with RDS in premature infants was detected. Analysis of rs2304566 polymorphisms using a logistic regression model showed that there are two significant predictors inversely related to the occurrence of RDS (Apgar score of 5 min, CT and TT genotype in rs2304566 polymorphism). Gestational age, birth weight, and sex have border significance. Moreover, in the patient group, the frequency of the GATGACA haplotype in the *SFTPB* gene was lower (*p* = 0.037), and the GATGGCA haplotype was higher (*p* = 0.059) in comparison with the control group.

**Conclusion:**

The common haplotype GATGACA of the *SFTPB* gene can be protective against RDS in preterm infants. The trend of a higher frequency of GATGGCA in the *SFTPB* gene in infants with severe RDS suggests that this haplotype may be a risk factor for RDS susceptibility.

## Introduction

The successful fetal-neonatal pulmonary transition depends on the appropriate production of pulmonary surfactant *in utero* (1). Surfactant deficiency may result in respiratory distress syndrome (RDS), which is the most common form of respiratory failure in newborn infants. Although the incidence of RDS is inversely related to gestational age, preterm delivery alone does not determine the risk of developing the disorder. Several studies have suggested the possible involvement of surfactant protein (SP) SP-A2 and SP-B, SP-C, and SP-D common genetic polymorphisms in the genetic predisposition to RDS (2–8).

Surfactant protein B is encoded by the *SFTPB* gene located on human chromosome 2p11.2 that spans 9.7 kb and encodes mature SP-B of 79-amino acids (9). SP-B plays a critical role in the intracellular processing of surfactant components, and it is essential for low surface tension in the alveoli (10). Pathogenic recessive mutations in *SFTPB* are associated with SP-B deficiency (11), known as pulmonary surfactant metabolism dysfunction type 1 (OMIM #265120). Until now, more than 50 different disease-causing mutations, resulting in acute respiratory distress in full-term infants, have been identified enabling both prenatal and postnatal genetic diagnosis (12, 13). This very rare condition is progressive and usually fatal at 3–6 months of age.

However, common DNA polymorphisms of the *SFTPB* gene have been identified in neonatal RDS. A variable short tandem repeat (STR) region in intron 4 of SP-B was analyzed, and higher rates of variant alleles in populations of infants with RDS were found when compared with controls (14, 15). A functional study demonstrated the involvement of this STR in *SFTPB* mRNA splicing (15). In addition, it was also reported that a common non-synonymous single-nucleotide polymorphism (SNP) variant rs1130866 is associated with RDS in preterm newborns (6, 16). Therefore, *SFTPB* is considered a candidate gene for neonatal RDS, and the role of common variants has to be elucidated.

In our previous studies, we have identified a risk haplotype comprising common alleles that were associated with fetal loss in women with sticky platelet syndrome (17). Additionally, we have identified common missense variant haplotypes involved in this condition (18). Thus, it appears that certain haplotypes may be associated with a functional effect on gene expression. Since disease-causing variants in the *SFTPB* gene are loss-of-function variants, we assume in RDS that common variants may have minimal effect on protein function or gene expression that is not phenotypically manifested in the later life of the neonate. To confirm our assumption, we aimed to explore haplotypes of common tagged SNPs in order to evaluate the hypothesis that certain haplotypes of *SFTPB* may be protective or risk factors for RDS.

## Material and Methods

### Study Group

Preterm infants (*n* = 149) born at University Hospital Martin and admitted to the Clinic of Neonatology were enrolled in the study. The major inclusion criteria were as follows: (1) gestational age under 33^rd^ + 6 weeks of gestation and (2) severity of respiratory distress. The newborns without or with mild respiratory distress were enrolled in the control group (*n* = 86); the infants with severe respiratory distress were included in the patient group (*n* = 63). Respiratory distress was considered severe if multiple doses of exogenous surfactant and/or mechanical ventilation within the first 72 h of life were needed. All the patients were Slovak and Caucasian. The exclusion criteria included severe perinatal asphyxia, early-onset sepsis, and congenital malformations.

The diagnosis of respiratory distress syndrome was based on the presence of clinical symptoms (tachypnea, dyspnea, retraction of jugulum, intercostal spaces and epigastrium, and cyanosis), X-ray findings with evidence of diffuse lung shading, and the need for ventilation support (19). All procedures related to postnatal stabilization and subsequent treatment were performed in accordance with the European consensus guidelines on the management of neonatal RDS in preterm infants (19, 20). The stabilization of the newborns was initiated in the delivery room by nasal continuous positive airway pressure (CPAP) applied through a face mask or short nasal prongs by T-resuscitator (Neopuff^®^) using a positive end-expiratory pressure (PEEP) of 6–8 cm H_2_O. Oxygen administration was adjusted according to pre-ductal saturation obtained by pulse oximetry. When indicated, the exogenous porcine surfactant (Curosurf, Chiesi Farmaceutici SpA, Parma, Italy) in 100–200 mg/kg doses was administered by INSURE or LISA methods. Subsequently, neonates were managed by non-invasive respiratory support either by nasal continuous positive airway pressure (CPAP) or nasal biphasic positive airway pressure (nBiPAP) (InfantFlow®). Indications for mechanical ventilation were apnoeic episodes and respiratory insufficiency despite the appropriate non-invasive respiratory support. Mechanical ventilation was performed in the pressure support ventilation (PSV+VG) mode. A second dose and, rarely, a third dose of surfactant were given when there was ongoing evidence of RDS, hypercarbia, high oxygen requirement, and other causative problems have been excluded. Gestational age, birthweight, gender, Apgar score, antenatal steroids, and preterm rupture of membranes (PROM) were recorded and assessed.

### DNA Isolation

The sample of venous blood (0.5 ml) was drawn into the EDTA tube. The DNA was extracted from blood samples at a volume of 0.2 ml by using the DNeasy Blood and Tissue DNA Isolation Kit (Qiagen, Germany) according to the manufacturer's instructions. DNA samples were diluted to 20 ng/μl and stored at −20°C before further processing.

### Selection of Tagged SNPs in the *SFTPB* Gene

**Figure 1:**
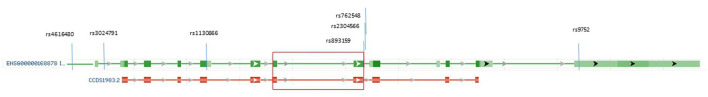
The reference transcript for the *SFTPB* gene (green section) and the SP-B preprotein (red section). The *SFTPB* gene reference transcript ENSG00000168878 is composed of 11 exons, with dark green rectangles aligning the exons that encode the SP-B preprotein, which is composed of 381 amino acid residues (CCDS1983.2, red section below transcript). The mature SP-B protein contains amino acid residues at codons 201 to 279 (28, 29), and protein-encoding exons 6 and 7 are indicated by a large red rectangle. Three of the tagged SNPs (rs893159, rs2304566, and rs762548) are in the second exon encoding mature SP-B, and SNP rs9752 is at 3′UTR in the flanking arm of the preprotein (light green transcript rectangle). https://www.ncbi.nlm.nih.gov/gene/6439 (modified).

In total, seven tagged SNPs (tagSNPs), such as rs9752, rs762548, rs2304566, rs893159, rs1130866, rs3024791, and rs4616480, were chosen directly in or near of *SFTPB* gene (chromosome 2, position from 85659544 to 85670645 according GRCh38.p7) and are characterized in Table 1. For the tag selection, we used Hap Map Data Rel 27/phase II + III on the NCBI B36 assembly, dbSNP b126 with minor allele frequency MAF > 0.1 in the Caucasian population, and Haploview software (Broad Institute, USA) applying the r^2^ threshold of 0.8. The distribution of tagged SNPs is shown in Figure 1.

**Table 1:** Characterization of tagged SNPs.

**SNP**	**Chromosome**	**HGVS name**
rs9752	2	NC_000002.12:g.85659544G>C
rs762548	2	NC_000002.12:g.85663630A>G
rs2304566	2	NC_000002.12:g.85663635T>C
rs893159	2	NC_000002.12:g.85663648T>G
rs1130866	2	NC_000002.12:g.85666618G>A
rs3024791	2	NC_000002.12:g.85668581C>T
rs4616480	2	NC_000002.12:g.85670645G>A

### Genotyping by Allelic Discrimination

DNA genotyping of tagged SNPs was performed by allelic discrimination. We used the commercially available TaqMan Universal PCR Mastermix (Applied Biosystems, USA) and TaqMan SNP Genotyping Assays (Applied Biosystems, USA) C_8696006_10 for rs9752, C_8695999_20 for rs893159, C_15984495_10 for rs3024791, and C_30785447_10 for rs4616480. The PCR was performed with 20 ng of DNA in a 20 μl volume according to the protocol of the manufacturer. Briefly, 10 μl of the 2 × concentrated TaqMan Universal Master Mix (Applied Biosystems, USA), 0.5 μl of the 40 × concentrated, or 1 μl of the 20 × concentrated appropriate TaqMan SNP Genotyping Assay (Applied Biosystems, USA), and 1 μl of the diluted DNA were amplified for 2 min at 50°C, 10 min at 95°C, and 40 times at 92°C for 15 s, and 60°C for 1 min. The genotypes were evaluated from the allelic discrimination plot using software version 2.0.1.

### Genotyping by High Resolution Melting Analysis (HRMA) and Confirmation by Dideoxysequencing

For the rs762548, the HRMA was performed in a 15 μl reaction with forward primer 5′-GCGCTGGCCCAAGTGA-3′ and reverse primer 5′- CATGCAGTGGCAGGGTAGG-3′ using 7.5 μl LighCycler 480 High Resolution Melting Master (Roche, Switzerland), 3 mM final concentration of Mg^2+^, 0.25 μM of each forward and reverse primer, and 15 ng of template DNA. The annealing temperature was 60°C, and the size of the amplified PCR products was 79 bp. In 10% of cases, the genotype was confirmed by dideoxysequencing using the forward primer as for HRMA and the reverse primer 5′-GCCACCCTACAGAGGTGTTT-3′. The PCR for sequencing was performed with 100 ng of extracted DNA in a final volume of 20 μl using FastStart Taq polymerase (Roche, Switzerland) with a 1.5 mM of Mg^2+^ concentration. The PCR conditions were 10 min at 95°C and 30 cycles at 95°C for 30 s, appropriate annealing temperature for 30 s, and 72°C for 30 s. The presence of PCR products was verified by using 1.75% agarose gel electrophoresis. Subsequently, PCR products were purified with the NucleoSpin Extract II kit (Machery-Nagel, UK), and the sequencing reaction was performed using the BigDye Terminator kit version 1.1 (Applied Biosystems, USA) at 95°C for 2 min and 35 times at 95°C for 15 min and 60°C for 4 min. The fragments were separated on the automatic genetic analyzer AB3500 and subsequently evaluated with DNA Sequencing Analysis Software version 5.4 (Applied Biosystems, USA) and ChromasPro version 2.4.4 (Technelysium, Australia).

### Statistical Analysis

Hardy–Weinberg equilibrium (HWE), genotype, allele, haplotype frequencies, and linkage disequilibrium between the gene polymorphisms were analyzed by using SHEsis software (http://shesisplus.bio-x.cn/SHEsis.html). Haplotype analysis was done using SVS 8 software (SNP and Variation Suite version 8.1, Golden Helix, Bozeman, MT).

Pearson's chi-square test was used for univariate comparisons of allele and genotype frequencies between the patient group and control group and for checking for deviation from HWE. Odds ratios (OR) with 95% confidence intervals (95% CI) were calculated to identify the relationship between SNPs and RDS.

Epi Info 7 was used for the rest of the analysis. Continuous variables were expressed as mean ± SD and compared by *t*-test, while the binomial variables were tabulated and compared with the chi-square test using Yates's correction.

Multivariate logistic regression analysis and the odds ratio were calculated to detect the contribution of the studied variables to the development of RDS. The following variables were included in the logistic model: gestational age, Apgar score of 5 min, birth weight, sex, antenatal steroids, and rs2304566 (model 1) or rs9752 (model 2). The value of *p* < 0.05 was regarded as statistically significant.

## Results

### Clinical Description of the Study Population

The main characteristics of both groups are summarized in Table 2. The patient and control groups differed significantly by gestational age, birthweight, and postnatal adaptation is expressed by Apgar scores.

**Table 2:** Characterization of enrolled subjects.

**Variable**	**Patients (*n* = 63)**	**Control (*n* = 86)**	***P*** **-value**
Gestational age (weeks)	27.2 ± 2.7	30.6 ± 2.0	<0.001
Birth weight (g)	1001.8 ± 393.2	1526.7 ± 391.7	<0.001
1 min Apgar score	4 [0–9]	6 [3–9]	<0.001
5 min Apgar score	6 [2–8]	7 [5–10]	<0.001
Male gender	34 (54.0%)	44 (51.2%)	0.8628
Preterm rupture of membranes	13 (20.6%)	19 (22.1%)	0.9902
Antenatal steroids	36 (57.1%)	37 (43.0%)	0.1012

*Gestational age and birth weight are displayed as mean ± SD; Apgar score is shown as median and the interval; and other variables are shown as number of subjects and percentage*.

### Analysis of Individual Polymorphisms in *SFTPB* Gene

The comparison of individual polymorphisms localized in the *SFTPB* gene in patients (*n* = 63) and control (*n* = 86) groups is shown in Table 3. Based on a calculation of Hardy–Weinberg equilibrium, the distribution of genotypes is consistent with control and patient groups (*p* > 0.05). Using a general genetic model for the estimation of associations between genotypes and RDS, no significant correlation was found. However, when we analyzed the distribution of single alleles (Table 4), we could show that the frequency of the C (minor) allele for rs9752 was significantly higher in the patient group compared to the control group (0.373 vs. 0.261) (*p* = 0.039, OR 1.679, 95% CI: 1.022–2.757). Similarly, the C (minor) allele of rs2304566 was significantly more frequent in the patient group compared with controls (0.269 vs. 0.171) (*p* = 0.039; OR 1.796, 95% CI: 1.025–3.148). For the other SNPs, only insignificant differences in the allele frequencies between the two groups were present (*p* > 0.05). After the false discovery rate was controlled at a level of 0.05 (Benjamini–Hochberg method), none of the differences in allele frequencies remained significant.

**Table 3:** Comparison of gene polymorphisms (SNPs) in the *SFTPB* gene.

**SNP**	**Group**	**Genotype a genotype frequency**			***P*** **-value**
rs9752		G/G	G/C	C/C	
	patient	27(0.428)	25(0.396)	11(0.174)	0.159
	control	49(0.569)	29(0.337)	8(0.093)	
rs762548		A/A	A/G	G/G	
	patient	54(0.857)	8(0.126)	1(0.015)	0.496
	control	75(0.882)	10(0.117)	0(0)	
rs2304566		T/T	T/C	C/C	
	patient	36(0.571)	20(0.317)	7(0.111)	0.16
	control	60(0.705)	21(0.247)	4(0.047)	
rs893159		T/T	T/G	G/G	
	patient	1(0.015)	10(0.158)	52(0.825)	0.725
	control	1(0.011)	18(0.209)	67(0.779)	
rs1130866		G/G	G/A	A/A	
	patient	12(0.19)	33(0.523)	18(0.285)	0.382
	control	14(0.162)	38(0.441)	34(0.395)	
rs3024791		C/C	C/T	T/T	
	patient	51(0.809)	11(0.174)	1(0.015)	0.355
	control	75(0.872)	11(0.127)	0(0)	
rs4616480		G/G	G/A	A/A	
	patient	17(0.269)	32(0.507)	14(0.222)	0.674
	control	29(0.337)	39(0.453)	18(0.209)	

**Table 4:** Distribution of individual genotypes in gene *SFTPB*.

**SNP**	**Group**	**Genotype frequency**		**P-** ** value**	**OR** **(95% CI)**	**HWE** **(patient)** ***P*****-value**	**HWE** **(control)** ***P*****–value**
rs9752		G	C				
	patient	79 (0.626)	47 (0.373)	0.039	1.679	0.484	0.498
	control	127 (0.738)	45 (0.261)		(1.022–2.757)		
rs762548		A	G				
	patient	116 (0.92)	10 (0.079)	0.486	1.379	0.582	0.847
	control	160 (0.941)	10 (0.058)		(0.556–3.421)		
rs2304566		T	C				
	patient	92 (0.73)	34(0.269)	0.039	1.796	0.304	0.504
	control	141 (0.829)	29 (0.17)		(1.025–3.148)		
rs893159		T	G				
	patient	12 (0.095)	114 (0.904)	0.562	0.8	0.821	0.985
	control	20 (0.116)	152 (0.883)		(0.375–1.703)		
rs1130866		G	A				
	patient	57 (0.452)	69 (0.547)	0.234	1.326	0.902	0.83
	control	66 (0.383)	106 (0.616)		(0.832–2.115)		
rs3024791		T	C				
	patient	13 (0.103)	113 (0.896)	0.219	1.683	0.904	0.818
	control	11 (0.063)	161 (0.936)		(0.728–3.893)		
rs4616480		G	A				
	patient	66 (0.523)	60 (0.476)	0.491	1.175	0.989	0.77
	control	97 (0.563)	75 (0.436)		(0.741–1.865)		

### Haplotype Estimation of Tagged SNPs in *SFTPB* Gene

Haplotype analysis was performed on seven tagged SNPs. The most frequent haplotypes are shown in Table 5. The frequency of haplotype GATGACA (in the following order: rs9752, rs762548, rs2304566, rs893159, rs1130866, rs3024791, and rs4616480) was lower in neonates with severe respiratory symptoms compared to neonates with no or only mild respiratory symptoms (0.126 vs. 0.223); the difference was statistically significant (*p* = 0.037; OR 0.512, 95% CI: 0.271–0.969). A similar trend close to statistical significance (*p* = 0.073; OR 0.427, 95% CI: 0.164–1.11) was present in the distribution of haplotype GATGACG. Its presence in the patient group was lower than in the control group (0.047 vs. 0.105). On the contrary, the frequency of haplotype GATGGCA was higher in the patient group than in the control group (0.15 vs. 0.082) (*p* = 0.059, OR 2.004, 95% CI: 0.963–4.169).

**Table 5:** Comparison of haplotypes in gene *SFTPB*.

**Haplotype**	**Patients** **(frequency)**	**Control** **(frequency)**	**Chi^**2**^**	**Fisher** **p-value**	**Pearson** **p-value**	**OR** **[95% CI]**
CACGACA	20 (0.158)	20 (0.117)	1.127	0.306	0.288	1.433 [0.735~2.795]
GATGACA	16 (0.126)	38 (0.223)	4.326	0.047	0.037	0.512 [0.271~0.969]
GATTACG	9 (0.071)	12 (0.07)	0.003	1	0.955	1.025 [0.418~2.514]
GATTGCG	3 (0.023)	6 (0.035)	0.304	0.738	0.581	0.674 [0.165~2.751]
GATGGCA	19 (0.15)	14 (0.082)	3.556	0.064	0.059	2.004 [0.963~4.169]
GATGGCG	20 (0.158)	32 (0.188)	0.376	0.643	0.539	0.825 [0.447~0.523]
GATGACG	6 (0.047)	18 (0.105)	3.194	0.086	0.073	0.427 [0.164~1.11]
CGTGACG	4 (0.031)	5 (0.029)	0.017	1	0.893	1.095 [0.288~4.162]
CATGACG	3 (0.023)	8 (0.047)	1.054	0.366	0.304	0.5 [0.129~1.923]

### Multivariate Logistic Regression Analysis

Analysis of rs2304566 polymorphisms (model 1) using a logistic regression model showed that there are two significant predictors inversely related to the occurrence of RDS (Apgar score of 5 min, CT and TT genotype in rs2304566 polymorphism). Moreover, gestational age, birth weight, and sex have borderline significance (Table 6).

**Table 6:** Logistic regression analysis (model 1).

**Variable**	**Odds Ratio**	**Lower limit (95% CI)**	**Upper limit (95% CI)**	**Coefficient**	**S.E**.	**Z-Statistic**	***P*** **-Value**
Gestational age	0.7372	0.5422	1.0024	**–**0.3049	0.1568	**–**1.9445	0.0518
Apgar in 5th minute	0.4017	0.2485	0.6491	**–**0.9121	0.2449	**–**3.7246	0.0002
Birth weight	0.9982	0.9963	1.0001	**–**0.0018	0.001	**–**1.8248	0.068
Gender—male (ref. female)	2.6192	0.921	7.4484	0.9629	0.5332	1.8056	0.071
Antenatal steroids	2.0253	0.7477	5.4861	0.7057	0.5084	1.3881	0.1651
**rs2304566 - C/T (ref C/C)**	**0.1295**	**0.0184**	**0.9105**	**−2.0441**	**0.9951**	**−2.0541**	**0.04**
**rs2304566 - T/T (ref C/C)**	**0.0709**	**0.0107**	**0.4704**	**−2.6468**	**0.9656**	**−2.7411**	**0.0061**
CONSTANT				18.4075	4.3608	4.2211	<0.0001

*SE, standard error; CI, confidence interval*.

After including polymorphism rs9752 (model 2, results not shown) into the logistic regression model, it was found that this polymorphism is not associated with RDS.

## Discussion

The etiology of RDS is multifactorial, and the phenotype of the RDS is likely influenced by several genetic factors, the proportion of which is estimated between 35 and 65% (21). The incidence and severity are inversely proportional to gestational age. Based on Vermont Oxford Network data in 2017, the incidence of RDS is about 80% in babies born at the 28th week of gestation increasing to 90% at the 24th week of gestation (19). The lower gestational age in the patient group in our study, where only infants with severe RDS were included, is consistent with this data. The differences between the patient and control groups in birth weight and postnatal adaptation expressed by the Apgar score can be attributed to lower gestational age. The literary data supports this assumption (22). Antenatal steroids as a protective factor and male gender as a risk factor can affect the course of RDS (23, 24), but the patient and control groups in our study did not differ in these variables.

The aim of our study was to explore the association of *SFTPB* gene haplotypes that were derived from a common SNP with a MAF > 0.1. We also analyzed all 7 tagged SNPs spanning the *SFTPB* gene separately in all enrolled subjects. The statistical evaluation of each SNP individually did not reveal any significant association with RDS in our cohort, and there were no differences observed between the patients and controls, as we expected. Nevertheless, we found an intronic TT genotype in the rs2304566 polymorphism to be protective of severe RDS, but its functional impact is not known yet. The second SNP where we found a significant association with RDS is rs9752. SNP rs9752 is located at 3′UTR. Recently, the association of recessive homozygotes harboring the CC genotype and heterozygotes with the CT genotype has been demonstrated with reduced plasma pro-SFTPB in non-small cell lung cancer patients (25), which could also indicate some functional effects of this SNP on gene expression.

The missenses SNP rs1130866 (Thr131Ile) was one of the included SNPs in this analysis; however, the association with RDS was not confirmed. Contrary to our result, an association between the SP-B Ile131Thr polymorphism and RDS was found in the twin study. The authors conclude that the concordance difference between monozygotic and dizygotic twin pairs underestimates the genetic impact on the risk of RDS (16). In the Greek population, no association between the SP-B Ile131Thr polymorphism and RDS was found (6).

A very recent study used 17 well-studied SNPs of *SFTPA1, SFTPA2, SFTPB, SFTPC*, and *SFTPD* to investigate the impact of individual SNPs and their interactions among SNPs in the same gene or between two or among three different genes on RDS. The authors show not only the association of a single SNP but also of two and three SNP interactions associated with RDS susceptibility. In the *SFTPB* gene, there was one significant intragenic interaction (rs2077079, rs3024798, and rs7316). Each SNP exhibited a dominant effect, and this interaction was associated with a decreased risk of RDS (2).

In our study, genotyping followed by a comparison of the *SFTPB* polymorphisms by Hardy–Weinberg equilibrium revealed a consistent distribution of different genotypes in both groups, namely controls and patients. However, for rs230456 polymorphisms, the minor allele C frequency was significantly higher in patients than in the control group.

We found out that the haplotype GATGACA (in the following order: rs9752G, rs762548A, rs2304566T, rs893159G, rs1130866A, rs3024791C, and rs4616480A) in the *SFTPB* gene showed significantly lower frequency in preterm infants with severe RDS compared to controls. The rs2304566 minor allele C associated with RDS in preterm neonates, as mentioned above, is not present in this haplotype. A similar trend, close to statistical significance, was observed in haplotype GATGACG (rs in the above-mentioned order) distribution. Its frequency in the patient group was lower than in the control group. The rs2304566 is here also represented by major allele T. Taken together, these two haplotypes can be considered as protective factors against the development of RDS in preterm infants. To the best of our knowledge, only one study analyzing SP-B haplotypes based on common SNPs in association with RDS has been published. However, this study used the family-based association test (FBAT) and extended transmission disequilibrium test (ETDT) to test −18 A/C (rs2077079 g.85668215T>C), 1,013 A/C (rs3024798 g.85667185G>T), 1,580 C/T (rs1130866 g.85666618G>A; Thr131Ile), and 9,306 A/G (rs7316 g.85658890T>C). By FBAT, two protective haplotypes have been identified and, in both cases, the rs1130866 was present as the Ile allele (3). In protective haplotypes in our study, the rs1130866 is also present as the Ile allele.

On the contrary, the haplotype GATGGCA in the patient group tended to be higher than in the control group. It means that the probability of the occurrence of RDS in exposed individuals is higher, and thus this haplotype can be identified as a risk factor for the development of RDS. In this case, the rs1130866 is present as the Thr allele. For that reason, the role of rs1130866 in the development of RDS in preterm neonates remains to be elucidated.

We suggest that other genetic and epigenetic factors can play a modifying role, or in certain populations, the rs1130866 can be linked to the locus affecting the expression of the *SFTPB* gene, and therefore, the results for rs1130866 are still controversial. Glycosylation of pro-SP-B or genetic polymorphisms within the promoter region has a role in genetic susceptibility to RDS. In a mice model, SP-B levels were correlated with the Ile131Thr genotype, and the lowest SP-B levels were observed in Thr/Thr homozygotes. This rs1130866 variation-dependent N-glycosylation was associated with the decreased levels of SP-B, which was secreted from fetal lung to amniotic fluid (26). Lower levels of pro-SP-B were also confirmed in human amniotic fluid samples and were associated with rs1130866 (9).

The results of our study contribute to a better understanding of the genetic background of RDS in preterm infants. The main strength of this study was the ethnically homogenous study group. The other advantages were the strict inclusion and exclusion criteria and homogenous management of obstetrical and neonatal care. On the contrary, the study had some limitations. It was conducted as a single-center study, and the number of participants is limited. The results should be replicated in future larger studies.

## Conclusion

At least one common haplotype GATGACA of the *SFTPB* gene can protect preterm newborns from RDS. The trend of higher frequency of GATGGCA in the *SFTPB* gene in preterm infants with severe respiratory distress suggests that this haplotype may be a risk factor for the occurrence of RDS. However, other genetic factors must be elucidated as candidate genes for the genetic susceptibility for RDS in preterm neonates. Further research is required to clarify the exact mechanism by which these genetic variants predispose to neonatal RDS.

Common SNPs tagged to a haplotype have been shown to have some protective and risk associated with RDS in premature newborns, emphasizing the genetic component of RDS. However, this association is weak and rather indicates a linkage disequilibrium of a certain haplotype or SNP with rare alleles in some patients. However, in the study design, we only concentrated on MAF > 0.1 and thus could not identify rare alleles. Sequencing analyses of whole genes in premature infants have identified novel rare variants associated with RDS. These are mostly in-frame deletions, missense synonyms, and variants in introns and 3′UTR, and according to the ACMG-AMP criteria, these are classified as variants of unknown significance (27). Therefore, it is necessary for the future to focus on the sequencing of whole genes and the identification of rare variants, which cannot be captured by haplotypes with common SNPs. Functional studies and patient follow-up can further identify the effect of these variants on the protein function and disease course, respectively.

## Data Availability Statement

The data that support the findings of this study are available on request from the corresponding authors, ZL and KM.

## Ethics Statement

The studies involving human participants were reviewed and approved by Ethics Committee of Jessenius faculty of Medicine, Comenius University registered under no. IORG0004721 at the US Office for Human Research Protection, US Department of Health and Human Services, and certified with the code IRB00005636 Jessenius Faculty of Medicine, Comenius University in Martin IRB # 1. Written informed consent to participate in this study was provided by the participants' legal guardian/next of kin.

## Author Contributions

SM conceptualized the study, wrote the study protocol, included the patients, collected the data, and wrote the paper. ZL conceptualized the study, wrote the study protocol, and performed the final interpretation of the results. VH performed processing and analysis of biological samples. MS was engaged in the analysis of haplotypes. JZ performed the statistical analyses. KM supervised the study and participated in the interpretation of the results. MZ and AC designed the study, oversaw the data analysis and interpretation, and critically reviewed and revised the manuscript drafts. All authors contributed substantially to the writing of the manuscript. All authors read and approved the final manuscript.

## Funding

This publication was created with the support of the Operational Programme Integrated Infrastructure for the project: Research and development in the medical sciences, the pathway to personalized treatment of serious neurological, cardiovascular, and cancer diseases, ITMS: 313011T431, cofinanced by the European Regional Development Fund and project APVV-17-0250.

## Conflict of Interest

The authors declare that the research was conducted in the absence of any commercial or financial relationships that could be construed as a potential conflict of interest.

## Publisher's Note

All claims expressed in this article are solely those of the authors and do not necessarily represent those of their affiliated organizations, or those of the publisher, the editors and the reviewers. Any product that may be evaluated in this article, or claim that may be made by its manufacturer, is not guaranteed or endorsed by the publisher.
